# Enhancing Kidney Transplantation Outcomes Through Robotic‐Assisted Paired Kidney Exchange: A Case Report

**DOI:** 10.1155/crit/9302274

**Published:** 2026-03-08

**Authors:** Vitor Turra, Sarah Rombach, Joao Manzi, Simone Zaragoza, Giselle Guerra, Rodrigo Vianna, Phillipe Abreu

**Affiliations:** ^1^ Miami Transplant Institute, Jackson Memorial Hospital, University of Miami, Miami, Florida, USA, miami.edu

**Keywords:** ABO incompatibility, ESRD, HLA incompatibility, kidney transplant, paired exchange, transplant surgery, kidney transplant, paired kidney exchange, robotic surgery

## Abstract

Chronic kidney disease (CKD) is a significant global health challenge with a rising prevalence, primarily affecting adults with underlying diabetes and hypertension. End‐stage renal disease (ESRD), the most severe form of CKD, necessitates renal replacement therapy, such as dialysis or kidney transplantation, to sustain life. Although kidney transplantation offers superior survival and quality of life outcomes, the demand for donor kidneys far exceeds supply, leading to long waiting times and high mortality rates. Living donor kidney transplants demonstrate better outcomes, yet incompatibilities, such as blood type or human leukocyte antigen (HLA) sensitization, limit donor–recipient matches. Paired kidney exchange (PKE) is an innovative strategy that facilitates transplants between HLA‐incompatible or ABO‐incompatible donor–recipient pairs, increasing the donor pool and improving compatibility. This case report presents a PKE performed robotically at the Miami Transplant Institute, involving two incompatible pairs. Both donor and recipient surgeries were conducted using robotic‐assisted techniques, optimizing surgical precision and patient recovery. The robotic approach minimized complications, expedited recovery, and improved postoperative outcomes. This case underscores the potential of combining robotic surgery with PKE to address challenges in kidney transplantation, enhancing compatibility, and patient outcomes while reducing invasiveness and recovery time. Despite its promise, the widespread adoption of robotic‐assisted PKE is limited by cost, accessibility, and the need for robust support and educational systems to encourage donor participation.

## 1. Introduction

Chronic kidney disease (CKD) is a progressive and incurable condition that leads to high rates of morbidity and mortality, primarily affecting adults with underlying diabetes and hypertension. [1]. This condition is defined by a reduction in the kidney′s functional capacity, with an estimated glomerular filtration rate (eGFR) of less than 60 mL/min. Additionally, there must be markers of kidney injury or abnormalities detected through laboratory tests or imaging studies that persist for at least 3 months. [2].

CKD poses a significant global public health challenge, with a current prevalence estimated at approximately 13.4% (11.7%–15.1%) [3]. By the year 2040, CKD is projected to become the fifth leading cause of death worldwide, representing one of the highest mortality rates among diseases, which highlights the need for careful attention and intervention [4].

When the filtration rate decreases to below 15 mL/min per 1.73 m^2^, the condition is classified as end‐stage renal disease (ESRD). At this stage, kidney function is insufficient to sustain the survival of the patient [2]. Patients at this stage rely on renal replacement therapy either through chronic dialysis or kidney transplantation to improve survival [5]. Although most people with ESRD receive treatment via hemodialysis or peritoneal dialysis [2], kidney transplantation remains the standard of excellence in improving quality of life and expectancy [6, 7].

Approximately 56% of patients with ESRD who are on dialysis are actively waiting for a kidney transplant. However, there is a significant gap between the supply of available kidneys and the demand for transplants. As a result, waitlist times are increasing, and mortality rates are rising. Currently, only 25% of patients receive a transplant, whereas 6% die annually, and that number continues to grow. In the year 2022, a total of 4152 individuals in the United States died while awaiting a kidney transplant.

Research indicates that kidney transplants significantly enhance patient survival rates compared with dialysis. Furthermore, living kidney donations demonstrate superior outcomes relative to deceased donor transplants concerning both patient survival and allograft function [8]. Approximately one‐third of prospective living donors are determined to be incompatible with their intended recipients due to either blood group discrepancies or human leukocyte antigen (HLA) sensitization [9]. Therefore, it is crucial to expand the pool of living donors. Although incompatible transplants can be performed after desensitization, these cases face numerous challenges. These challenges include high costs and clinical issues, such as rejection and infection, which can lead to decreased survival rates for both the allograft and the patient. [10–12].

**Figure 1:**
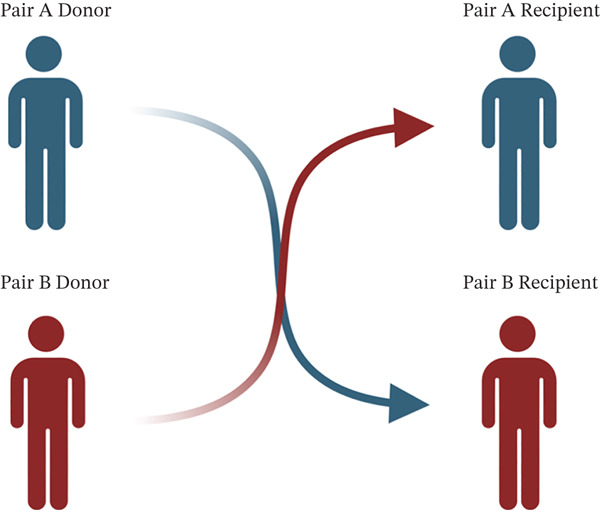
Paired kidney exchange involves switching donors to increase compatibility. This figure is created with http://BioRender.com.

In response to the need for more compatible living donor kidney transplants, both medical and surgical innovative solutions have been developed. One such strategy is paired kidney exchange (PKE), which involves exchanging kidneys between two or more living donor pairs that are either HLA‐incompatible or ABO‐incompatible. [13]. Typically, PKE is conducted as a bilateral exchange in which a donor from Pair A donates to a compatible recipient in Pair B, while simultaneously, a donor from Pair B donates to a compatible recipient in Pair A (refer to Figure 1). This exchange protocol can also be expanded to include three or more pairs or to facilitate the formation of chains. [14, 15].

Occasionally, compatible HLA and ABO pairs participate in PKE to improve the success rates of kidney transplants. These cases benefit from enhanced HLA compatibility, better‐sized organs, or younger donors [16]. To enhance the quality of care for both donors and recipients, surgical innovations, including robotics, have been implemented. This report presents a paired exchange case conducted by the Miami Transplant Institute (MTI) at Jackson Memorial Hospital. In this case, a compatible pair participated in the PKE program, and all procedures—both for donors and recipients—were performed robotically. This approach is aimed at achieving optimal survival rates and ensure the highest quality success for all involved.

## 2. Case Report

Pair A comprised a 35‐year‐old female patient requiring a kidney transplant due to ESRD caused by glomerulonephritis. She had been undergoing dialysis since August 2020 and had accumulated a waiting time of 18 months prior to the identification of a potential living donor. The designated donor was her 45‐year‐old husband, who had been medically evaluated and approved for donation; however, he was found to be incompatible due to a positive crossmatch, as the recipient exhibited sensitization with a calculated panel‐reactive antibody (cPRA) of 91%. Both the recipient and donor A have blood type O positive.

Pair B consisted of a 56‐year‐old male patient with Stage 4 CKD, presumably due to gout and hypertension. His potential donor was his 51‐year‐old wife, who was blood type compatible but had a mismatch in kidney size, characterized by her kidney being too small for the recipient, which would result in low nephron mass. Recipient B′s eGFR prior to transplantation was 17 mL/min/1.73 m^2^ with a creatinine level of 4.1, and he presented a cPRA of 0%. Both donors in Pair B, as well as the intended recipient, have blood type O positive.

All patients allowed personal data processing, and informed consent was obtained from all individual participants included in the study.

Consequently, through the utilization of electronic matching based on size and immunological parameters, both pairs were identified for participation in an exchange program. The living kidney donor team determined that all surgeries could be safely performed using robotic techniques. The procedures were conducted simultaneously within a 14‐h timeframe on March 30, 2023. Both donor procedures utilized robotic surgery via a Pfannenstiel incision. The perioperative and postoperative care was uneventful, with both donors being discharged home within 24 h, reporting minimal pain and no complications. Donor A donated her left kidney, whereas Donor B donated his right kidney, which necessitated some reconstruction of the renal vein length using a vessel from a deceased donor.

The transplant procedures for the recipients were also executed robotically without complications, resulting in Recipient A being discharged within 3 days, whereas Recipient B was discharged after 4 days. Similar to the donors′ surgeries, both transplanted kidneys were inserted into the abdominal cavities through a Pfannenstiel incision. As of the present date, all four patients are clinically stable. The follow‐up periods indicate posttransplant eGFR levels of 62, 50, and 60 for Recipient A and 50, 50, and 54 for Recipient B at 1, 3, and 6 months, respectively.

## 3. Discussion

The successful implementation of robotic surgery in PKE donations, as demonstrated in this case report from Jackson Memorial Hospital in Miami, represents a meaningful advancement in transplant surgery. Although the outcomes described are specific to a single case and should be interpreted with caution, the use of robotic‐assisted surgery for both donors and recipients within a PKE framework highlights the ongoing evolution of surgical strategies aimed at minimizing invasiveness, shortening recovery time, and optimizing perioperative outcomes [1–4].

The adoption of robotic surgery in kidney transplantation reflects a broader trend toward leveraging advanced technology to enhance surgical precision and patient safety. Robotic‐assisted approaches offer advantages such as improved dexterity, superior three‐dimensional visualization, and reduced ergonomic strain for surgeons [5–7]. In this case, the robotic platform facilitated safe donor nephrectomy and recipient transplantation with minimal complications and rapid postoperative recovery. The use of a Pfannenstiel incision further contributed to reduced postoperative pain, lower risk of wound‐related complications, and improved cosmetic outcomes, reinforcing the patient‐centered benefits of this approach [8].

At the same time, laparoscopic donor nephrectomy continues to demonstrate favorable outcomes, including reduced operative times, lower costs, and shorter learning curves in certain settings [17]. Recent studies suggest that laparoscopic techniques can achieve outcomes comparable with robotic surgery, particularly in experienced centers, though with more limited visualization and instrument articulation. Therefore, the choice between robotic and laparoscopic approaches should be guided by institutional expertise, case complexity, and economic considerations, rather than a single universally preferred technique.

Beyond the surgical platform, the incorporation of PKE offers important clinical advantages that extend beyond resolving donor–recipient incompatibility. PKE expands the donor pool by enabling transplantation in patients facing blood group incompatibility, HLA sensitization, or suboptimal donor anatomy, while also allowing for improved immunologic matching across pairs [9–13]. Improved matching has been associated with lower rejection rates, better graft function, and enhanced long‐term graft survival. Additionally, PKE programs can reduce waiting times for transplantation, limit prolonged dialysis exposure, and improve overall patient survival and quality of life.

This case highlights the synergistic relationship between robotic surgery and PKE in advancing kidney transplantation. The combination of minimally invasive surgical techniques with optimized donor–recipient matching may enhance perioperative recovery while simultaneously improving immunologic and long‐term clinical outcomes. Nevertheless, the absence of long‐term follow‐up data and a control group limits definitive conclusions regarding the superiority of robotic‐assisted PKE over alternative approaches [14–16].

Several challenges remain. Robotic surgery continues to face cost‐effectiveness concerns when compared with laparoscopic or open techniques, particularly given the high capital and maintenance costs and limited accessibility in many healthcare systems. Similarly, the success of PKE programs depends on patient and donor willingness to participate, underscoring the need for robust educational initiatives, psychosocial support, and transparent informed consent processes. Ethical considerations surrounding participation of compatible donor–recipient pairs have been well described and remain essential to ensure equitable access and trust in these programs.

Emerging data suggest favorable long‐term outcomes following robotic‐assisted kidney transplantation, including improved graft survival and patient outcomes in selected cohorts . As these techniques continue to evolve, balancing innovation with cost, access, and equity will be critical to ensure broad applicability and sustainable adoption.

In conclusion, the integration of robotic surgery within PKE programs represents a promising and complementary strategy to address both surgical and immunologic challenges in kidney transplantation, with the potential to enhance patient‐centered outcomes when applied in appropriately selected settings.

## Funding

No funding was received for this manuscript.

## Disclosure

This work has not been published elsewhere and is not under consideration by any other journal. All authors have reviewed and approved the manuscript.

## Conflicts of Interest

The authors declare no conflicts of interest.

## Data Availability

Data sharing is not applicable to this article as no datasets were generated or analyzed during the current study.

## References

[1] Kalantar-Zadeh K. , Jafar T. H. , Nitsch D. , Neuen B. L. , and Perkovic V. , Chronic Kidney Disease, The Lancet. (2021) 398, no. 10302, 786–802. 10.1016/S0140-6736(21)00519-5 34175022

[2] Webster A. C. , Nagler E. V. , Morton R. L. , and Masson P. , Chronic Kidney Disease, The Lancet. (2017) 389, 1238–1252. 10.1016/S0140-6736(16)32064-5 27887750

[3] Lv J. C. and Zhang L. X. , Prevalence and Disease Burden of Chronic Kidney Disease, Renal fibrosis: mechanisms and therapies. (2019) 1165, 3–15. 10.1007/978-981-13-8871-2_1 31399958

[4] Foreman K. J. , Marquez N. , Dolgert A. , Fukutaki K. , Fullman N. , McGaughey M. , Pletcher M. A. , Smith A. E. , Tang K. , and Yuan C.-W. , Forecasting Life Expectancy, Years of Life Lost, and All-Cause and Cause-Specific Mortality for 250 Causes of Death: Reference and Alternative Scenarios for 2016–40 for 195 Countries and Territories, The Lancet. (2018) 392, no. 10159. 10.1016/S0140-6736(18)31694-5 PMC6227505 30340847

[5] Zarantonello D. , Rhee C. M. , Kalantar-Zadeh K. , and Brunori G. , Novel Conservative Management of Chronic Kidney Disease via Dialysis-Free Interventions, Current Opinion in Nephrology and Hypertension. (2021) 30, no. 1, 97–107. 33186220 10.1097/MNH.0000000000000670

[6] Hariharan S. , Israni A. K. , and Danovitch G. , Long-Term Survival After Kidney Transplantation, New England Journal of Medicine. (2021) 385, no. 8, 729–743. 34407344 10.1056/NEJMra2014530

[7] Watson C. J. E. and Dark J. H. , Organ Transplantation: Historical Perspective and Current Practice, British Journal of Anaesthesia. (2012) 108, 10.1093/bja/aer384, 2-s2.0-84855224498. 22194428

[8] Terasaki P. I. , Cecka J. M. , Gjertson D. W. , and Takemoto S. , High Survival Rates of Kidney Transplants from Spousal and Living Unrelated Donors, New England Journal of Medicine. (1995) 333, no. 6, 333–336, 10.1056/NEJM199508103330601, 2-s2.0-0029114163, 7609748. 7609748

[9] Segev D. L. , Gentry S. E. , Warren D. S. , Reeb B. , and Montgomery R. A. , Kidney Paired Donation and Optimizing the Use of Live Donor Organs, JAMA. (2005) 293, no. 15, 1883–1890, 10.1001/jama.293.15.1883, 2-s2.0-17144414075. 15840863

[10] de Weerd A. E. and Betjes M. G. H. , ABO-Incompatible Kidney Transplant Outcomes: A Meta-Analysis, Clinical Journal of the American Society of Nephrology. (2018) 13, no. 8, 1234–1243, 10.2215/CJN.00540118, 2-s2.0-85051417025. 30012630 PMC6086717

[11] Marfo K. , Lu A. , Ling M. , and Akalin E. , Desensitization Protocols and Their Outcome, Clinical Journal of the American Society of Nephrology. (2011) 6, no. 4, 922–936, 10.2215/CJN.08140910, 2-s2.0-79955560884. 21441131

[12] Axelrod D. , Lentine K. L. , Schnitzler M. A. , Luo X. , Xiao H. , Orandi B. J. , Massie A. , Garonzik-Wang J. , Stegall M. D. , Jordan S. C. , and Oberholzer J. , The Incremental Cost of Incompatible Living Donor Kidney Transplantation: A National Cohort Analysis, American Journal of Transplantation. (2017) 17, no. 12, 3123–3130, 10.1111/ajt.14392, 2-s2.0-85025449711. 28613436 PMC12404663

[13] Kher V. and Jha P. K. , Paired Kidney Exchange Transplantation – Pushing the Boundaries, Transplant International. (2020) 33, no. 9, 975–984, 10.1111/tri.13693, 32634850. 32634850

[14] Saidman S. L. , Roth A. E. , Sönmez T. , Ünver M. U. , and Delmonico F. L. , Increasing the Opportunity of Live Kidney Donation by Matching for Two- and Three-Way Exchanges, Transplantation. (2006) 81, no. 5, 773–782, 10.1097/01.tp.0000195775.77081.25, 2-s2.0-33645052740. 16534482

[15] Keizer K. M. , De Klerk M. , Haase-Kromwijk B. J. J. M. , and Weimar W. , The Dutch Algorithm for Allocation in Living Donor Kidney Exchange, Transplantation Proceedings. (2005) 37, no. 2, 589–591, 10.1016/j.transproceed.2004.12.096, 2-s2.0-17844404540. 15848466

[16] Cuffy M. C. , Ratner L. E. , Siegler M. , and Woodle E. S. , Equipoise: Ethical, Scientific, and Clinical Trial Design Considerations for Compatible Pair Participation in Kidney Exchange Programs, American Journal of Transplantation. (2015) 15, no. 6, 1484–1489, 10.1111/ajt.13218, 2-s2.0-84929359150, 25773372. 25773372

[17] Abreu P. , Kadri H. , Maffei R. , Hansen K. , Yoeli D. , Di Napoli M. , Yoshida C. , Rodriguez I. , Lyons S. , Giusti S. , Montague T. , Cooper J. , Choudhury R. , Conzen K. , Adams M. , Kennealey P. , Bak T. , Nydam T. , and Pshak T. , The Road To 100: Single Center Experience With The First 100 Consecutive Robotic Kidney Transplants, Journal of Robotic Surgery. (2026) 20, no. 1, 10.1007/s11701-026-03205-y. 41689721

